# Retraction: siRNA directed against c-Myc inhibits proliferation and downregulates human telomerase reverse transcriptase in human colon cancer Colo 320 cells

**DOI:** 10.1186/1756-9966-28-101

**Published:** 2009-07-16

**Authors:** Huang Hao, Yu Nancai, Fu Lei, Wei Xiong, Su Wen, Huang Guofu, Wu yanxia, Huang Hanju, Liu Qian, Xiao Hong

**Affiliations:** 1 Center of Experimental Medicine, Wuhan No.1 Hospital, Wuhan, 430022, PR China; 2 Department of Pathogentic Biology, Tongji Medical College, Huazhong University of Science and Technology, Wuhan, 430030, PR China; 3 Brain Research Center, University of British Columbia, Vancouver, BC, Canada

## Retraction

The corresponding author submitted this article [1] to *Journal of Experimental and Clinical Cancer Research *although this article had been accepted and previously published by *Cancer Biotherapy & Radiopharmaceuticals *[2]. The article was also received and subsequently accepted and published by *Nucleosides, Nucleotides and Nucleic Acids *[3]. Since it has been brought to the attention of all authors that duplicate submission and publication have taken place the decision has been made to retract the article published in *Journal of Experimental and Clinical Cancer Research*. The authors are deeply sorry for any inconvenience this may have caused to the editorial staff and readers.
